# Indole-3-Lactic Acid Modulates Feeding Behavior and Appetite-Related Signals in Weaned Piglets

**DOI:** 10.3390/ani16213381

**Published:** 2026-10-27

**Authors:** Keyi Chen, Xinghua Dong, Huixuan He, Yu Huang, Yong Su

**Affiliations:** Laboratory of Gastrointestinal Microbiology, Jiangsu Key Laboratory of Gastrointestinal Nutrition and Animal Health, College of Animal Science and Technology, Nanjing Agricultural University, Nanjing 210095, China; 9201510614@stu.njau.edu.cn (K.C.);

**Keywords:** indole-3-lactic acid, feeding behavior, weaned piglets, appetite regulation, gastrointestinal hormones, hypothalamic transcriptome

## Abstract

Gut microbiota-derived metabolites act as important mediators linking the gut microbiome with host physiological functions and participate in the regulation of metabolic homeostasis and behavior. However, whether the tryptophan metabolite indole-3-lactic acid (ILA) regulates feeding behavior in piglets and its potential central regulatory mechanisms remain unclear. In this study, 12 weaned piglets were used as a model to investigate the effects of ILA on feeding behavior regulation. The results showed that ILA supplementation did not significantly affect growth performance (*p* > 0.05), although final body weight, average daily gain, and average daily feed intake were numerically higher in the ILA group than in the control group. ILA also altered feeding behavior patterns, characterized by reduced daily feeding frequency (*p* < 0.05) and increased feeding rate (*p* = 0.053). Furthermore, ILA significantly increased serum concentrations of several appetite-related gastrointestinal hormones, including glucagon-like peptide-1, peptide YY, ghrelin, and cholecystokinin, and modified serum lipid metabolism-related parameters (*p* < 0.05). Hypothalamic transcriptomic analysis identified 240 differentially expressed genes in the ILA group compared with the CON group, with enrichment of genes involved in neural signaling and appetite regulation. These findings suggest that ILA may influence feeding behavior in weaned piglets, accompanied by numerical increases in feed intake and growth-related parameters, as well as changes in peripheral appetite-related gastrointestinal hormones and hypothalamic transcriptional responses.

## Simple Summary

Weaning is a critical stage in pig production, as piglets must adapt from milk to solid feed. Reduced feed intake during this period can impair growth and health. Indole-3-lactic acid is a microbial metabolite derived from tryptophan, but its role in feeding regulation after weaning is not well understood. In this study, we examined whether indole-3-lactic acid affects feed intake or feeding behavior in weaned piglets. Supplementation with indole-3-lactic acid did not significantly alter overall growth performance, although it numerically increased average daily feed intake, average daily gain, and final body weight. Indole-3-lactic acid modified feeding patterns, with piglets eating less frequently but at a faster rate, while total feeding time remained unchanged. In addition, indole-3-lactic acid altered several hormones and metabolic indicators related to feeding, as well as hypothalamic gene expression involved in appetite regulation. These findings suggest that indole-3-lactic acid may influence feeding behavior in weaned piglets and provide new evidence that gut microbial metabolites contribute to feeding regulation during the post-weaning period.

## 1. Introduction

Weaning represents a critical physiological transition during piglet growth and development. During this period, piglets transition from suckling to consuming solid feed, while also experiencing substantial changes in housing conditions and nutrient availability. These changes require adaptations in gastrointestinal structure, digestive function, and feeding behavior [1,2]. However, owing to the immaturity of the gastrointestinal tract, weaned piglets commonly exhibit reduced feed intake, impaired nutrient utilization, and retarded growth, which may negatively affect their subsequent growth performance and health [3,4,5,6]. Therefore, establishing and regulating feeding behavior after weaning are important for facilitating piglets’ adaptation to the post-weaning period.

The initiation and maintenance of feeding are regulated by multiple factors, including nutritional status, feeding motivation, and satiety signals, through interactions between peripheral and central regulatory systems [7]. The hypothalamus is a key brain region involved in integrating whole-body energy status and regulating feeding. The gastrointestinal tract also provides peripheral signals related to food intake and nutritional status through nutrient sensing, mechanosensation, and gastrointestinal hormones [8]. Mechanosensory and chemosensory receptors in the digestive system detect the amount of feed, energy, and specific nutrients present in the gastrointestinal tract. This information is largely transmitted to the central nervous system via the vagus nerve. Gastrointestinal hormones also play important roles in this process. Ghrelin, glucagon-like peptide-1 (GLP-1), cholecystokinin (CCK), and peptide YY (PYY) regulate hunger, satiety, and feeding behavior through different signaling pathways.

Previous studies have demonstrated the involvement of these signals in feeding regulation in pigs. Exogenous administration of PYY3-36 significantly reduced short-term feed intake in pigs under both ad libitum feeding and fasting–refeeding conditions [9]. In weaned piglets, exogenous ghrelin treatment increased feeding behavior during certain observation periods, although total feed intake was not significantly affected [10]. Further studies in growing pigs have shown that feeding frequency alters central ghrelin levels and hypothalamic signaling pathways. These changes are accompanied by alterations in food anticipatory activity. In addition, administration of a ghrelin antagonist reduced activity during the 4 h preceding feed delivery [11]. Collectively, these findings indicate that feeding in pigs is influenced by interactions between peripheral signals and central regulatory responses, affecting both feed intake and feeding behavior.

In recent years, the gut microbiota and their metabolites have been increasingly recognized as important signaling mediators linking the intestinal microbial ecosystem to host physiological functions [12]. Gut microorganisms utilize dietary nutrients to produce a variety of bioactive metabolites that can interact with intestinal epithelial cells, enteroendocrine cells, and the nervous system to regulate host metabolism and behavior. Tryptophan, an essential amino acid, serves not only as a substrate for protein synthesis but also as a precursor for the microbial generation of diverse indole derivatives, including indole-3-propionic acid (IPA), indole-3-acetic acid (IAA), and indole-3-lactic acid (ILA). These metabolites have been implicated in host immune regulation, the maintenance of intestinal homeostasis, and host metabolic regulation [13,14]. Tryptophan metabolism is closely associated with intestinal barrier integrity and mucosal immune homeostasis. Tryptophan-derived metabolites can influence intestinal epithelial function and immune responses, while disturbances in the gut microbiota and its metabolic activities may compromise intestinal barrier integrity and contribute to inflammatory processes [15,16]. These findings highlight the importance of the interactions between microbial tryptophan metabolism, intestinal health, and host immune regulation.

Among these metabolites, ILA has attracted increasing interest because of its reported intestinal protective, anti-inflammatory, and neuromodulatory properties [17,18,19,20,21,22]. Recent research using an activity-based anorexia mouse model has further explored the role of ILA in feeding-related regulation, reporting changes in feeding-related behaviors and hypothalamic gene expression following ILA administration [23]. In addition, our previous study further showed that ILA promotes GLP-1 secretion and facilitates the differentiation of intestinal stem cells into enteroendocrine cells. These findings suggest that ILA may influence intestinal endocrine function in pigs and support further investigation into its potential role in gastrointestinal signaling [24]. Collectively, these findings support further investigation into the potential role of ILA in feeding regulation. However, it remains largely unknown whether ILA, as a microbiota-derived signaling metabolite, regulates feeding behavior in animals and whether its effects are mediated by changes in peripheral nutrient signals and central neural regulatory networks.

We hypothesized that ILA would alter feeding behavior in weaned piglets and that these changes would be accompanied by alterations in peripheral appetite-related signals and hypothalamic gene expression. In this study, we investigated the potential role of the gut microbiota-derived metabolite ILA in the regulation of feeding in weaned piglets. We evaluated the effects of ILA supplementation on growth performance, feeding behavior, serum metabolic parameters, appetite-related gastrointestinal hormones, and hypothalamic transcriptional responses. This study aimed to preliminarily explore the peripheral and central responses associated with ILA supplementation.

## 2. Materials and Methods

### 2.1. Animals, Experimental Design, and Sampling

The animal experiment was conducted in Zhenjiang, Jiangsu Province, China. Feed was provided manually, and piglets had free access to feed and drinking water throughout the trial. Vaccinations were administered according to the facility’s routine vaccination schedule. Piglets were monitored daily for general health status, including activity, feed intake, and any observable clinical signs of illness. No medications or therapeutic treatments were administered during the experimental period.

**Figure 1:**
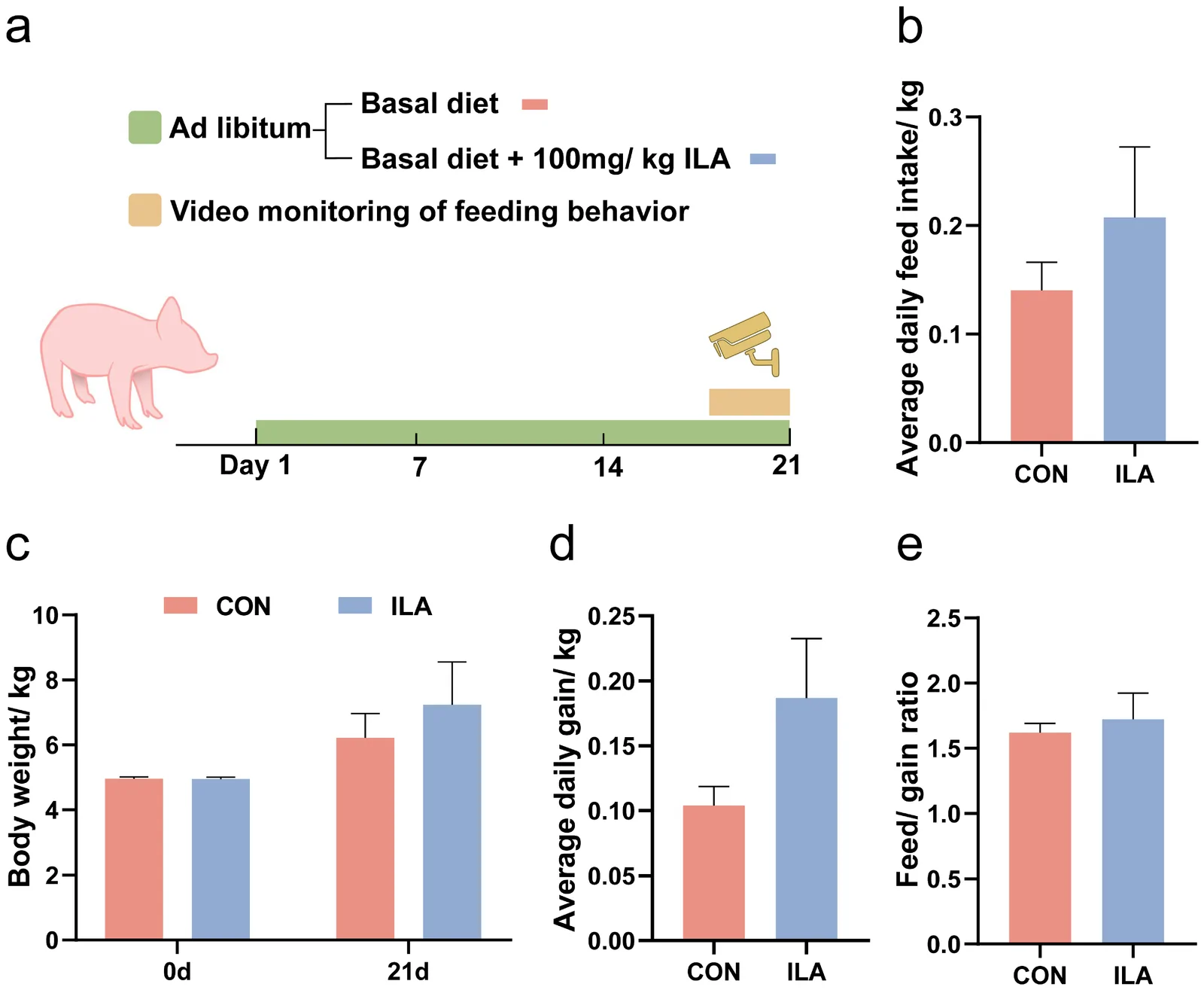
Effects of ILA on growth performance (*n* = 6): (**a**) Experimental procedure. (**b**) Average daily feed intake. (**c**) Body weight. (**d**) Average daily gain. (**e**) The ratio of feed and gain. Data are presented as the mean ± SEM. Abbreviations: CON, control group; ILA, indole-3-lactic acid.

Given the relatively high cost of ILA and the exploratory nature of this study, 12 healthy piglets (Duroc × Landrace × Large White), weaned at 21 ± 2 d of age with an average body weight of 4.95 ± 0.14 kg, were selected and randomly assigned to two dietary treatment groups (*n* = 6 per group): the control group (CON) and the ILA group (ILA). Piglets in the CON group received a basal diet, whereas those in the ILA group received the same basal diet supplemented with 100 mg/kg ILA. The basal diet (Table 1) was formulated to meet the nutrient requirements for piglets recommended by the NRC 2012 [25]. After a 3-day adaptation period, all piglets were subjected to a 21-day feeding trial (Figure 1a). ILA was purchased from Shanghai Macklin Biochemical Co., Ltd. (Shanghai, China) with a purity of 98%. In this experiment, ILA was supplemented into the basal diet at a concentration of 100 mg/kg. Before preparation of the experimental diet, ILA was premixed with a small amount of the basal diet and then thoroughly mixed with the remaining feed to facilitate uniform distribution. Piglets were individually housed and had free access to feed and water throughout the experimental period. Body weight was recorded on days 0, 7, 14, and 21 of the feeding trial, and feed intake was recorded daily to calculate average daily gain (ADG), average daily feed intake (ADFI), and feed-to-gain ratio (F/G). At the end of the feeding trial, all piglets were fasted overnight before sample collection. Blood samples were collected from the anterior vena cava into sterile vacuum tubes, which were immediately placed on ice (Hebei Kangweishi Medical Technology Co., Ltd., Shijiazhuang, China). Approximately 10 mL of blood was collected from the anterior vena cava of each piglet into sterile vacuum tubes and immediately placed on ice. Serum samples were obtained by centrifugation at 3000× *g* for 15 min at 4 °C, aliquoted, and stored at −20 °C until further analysis of serum biochemical parameters and gastrointestinal hormones. At the end of the feeding trial, the piglets were electrically stunned by trained slaughterhouse personnel and subsequently slaughtered. Following slaughter, the skull was opened, and the hypothalamus was carefully dissected from each piglet. The entire hypothalamus was collected and cut into small pieces, immediately frozen in liquid nitrogen, and stored at −80 °C until further analysis.

### 2.2. Definition of Feeding Behavior

Piglet behavior was continuously recorded using infrared video cameras for three consecutive days starting on day 19 of the feeding trial (Hikvision, Hangzhou, China). A feeding event was defined as occurring when a piglet kept its head above or inside the feeder for at least 5 s, or when chewing behavior was observed while its mouth was in direct contact with the feed. Total daily eating frequency referred to the total number of eating events recorded within each 24 h period. Total daily eating time represented the cumulative duration of all eating events within each 24 h period. Eating rate was expressed as total feed intake per minute of eating time (g/min). To characterize the distribution of eating behavior throughout the day, each 24 h period was divided into 24 consecutive 1 h intervals. Eating frequency for each interval represented the number of eating events occurring during that hour, whereas eating time represented the cumulative duration of all eating events occurring within that hour. The values for each piglet were averaged across the three consecutive recording days before statistical analysis.

### 2.3. Serum Biochemical Profiles

The following serum biochemical parameters were determined using an automatic biochemical analyzer (BK-280; BIOBASE, Jinan, China) according to the manufacturer’s instructions: glucose (GLU), alanine aminotransferase (ALT), aspartate aminotransferase (AST), total protein (TP), albumin (ALB), total bile acid (TBA), urea nitrogen (UN), cholesterol (CHO), triglycerides (TGs), high-density lipoprotein cholesterol (HDL-C), low-density lipoprotein cholesterol (LDL-C), and non-esterified fatty acids (NEFAs).

### 2.4. Determination of Serum Appetite-Related Gastrointestinal Hormones

The concentrations of serum gastrointestinal hormones, including GLP-1, PYY, ghrelin, CCK, and leptin (LEP), were determined using commercial enzyme-linked immunosorbent assay (ELISA) kits (mlbio, Shanghai, China) according to the manufacturer’s instructions.

### 2.5. Transcriptome Analysis of Hypothalamus

Total RNA was extracted from hypothalamic tissue using TRIzol reagent (Life Technologies, Carlsbad, CA, USA) according to the manufacturer’s instructions. RNA quality was assessed using NanoDrop 2000 (Thermo Fisher Scientific, Wilmington, DE, USA) and Agilent 2100 Bioanalyzer (Agilent Technologies, Santa Clara, CA, USA). Qualified RNA samples were used for mRNA library construction using the Hieff NGS Ultima Dual-mode mRNA Library Prep Kit for Illumina (Yeasen Biotechnology (Shanghai) Co., Ltd., Shanghai, China) and sequenced on an Illumina NovaSeq platform to generate paired-end 150 bp reads (Illumina, Inc., San Diego, CA, USA). After quality control, clean reads were aligned to the pig reference genome using Hisat2, and gene expression levels were quantified based on FPKM values. The differentially expressed genes (DEGs) between the control and ILA groups were identified using edgeR with the criteria of fold change ≥ 1.5 or ≤0.67 and *p*-value < 0.05. Kyoto Encyclopedia of Genes and Genomes (KEGG) pathway enrichment analysis was performed to identify signaling pathways associated with ILA-induced changes in hypothalamic gene expression.

### 2.6. Statistical Analysis

SPSS version 20.0 (IBM Corp., Armonk, NY, USA) and GraphPad Prism 8.0 (GraphPad Software, San Diego, CA, USA) were used for statistical analysis and graph plotting. Data are presented as mean ± SEM. The normality of the data distribution was evaluated using the Shapiro–Wilk test. For normally distributed variables, differences between the CON and ILA groups were assessed using an independent samples *t*-test. For variables that did not meet the assumption of normality, differences between groups were analyzed using the Mann–Whitney U test. Spearman’s rank correlation analysis was performed to evaluate the associations among hypothalamic appetite-related gene expression, feeding behavior parameters, serum biochemical profiles, and appetite-related hormone levels. Statistical significance was defined as *p* < 0.05.

## 3. Results

### 3.1. ILA Did Not Affect Growth Performance

Following the 21-day feeding trial, ILA did not significantly affect growth performance parameters, including final body weight (BW), average daily gain (ADG), average daily feed intake (ADFI), and feed-to-gain ratio (F/G), compared with the control group (Figure 1b–e). Although no significant differences were observed, the ILA group showed numerically higher final BW, ADG, and ADFI than the control group.

### 3.2. ILA Altered Serum Lipid Metabolic Profiles

**Figure 2:**
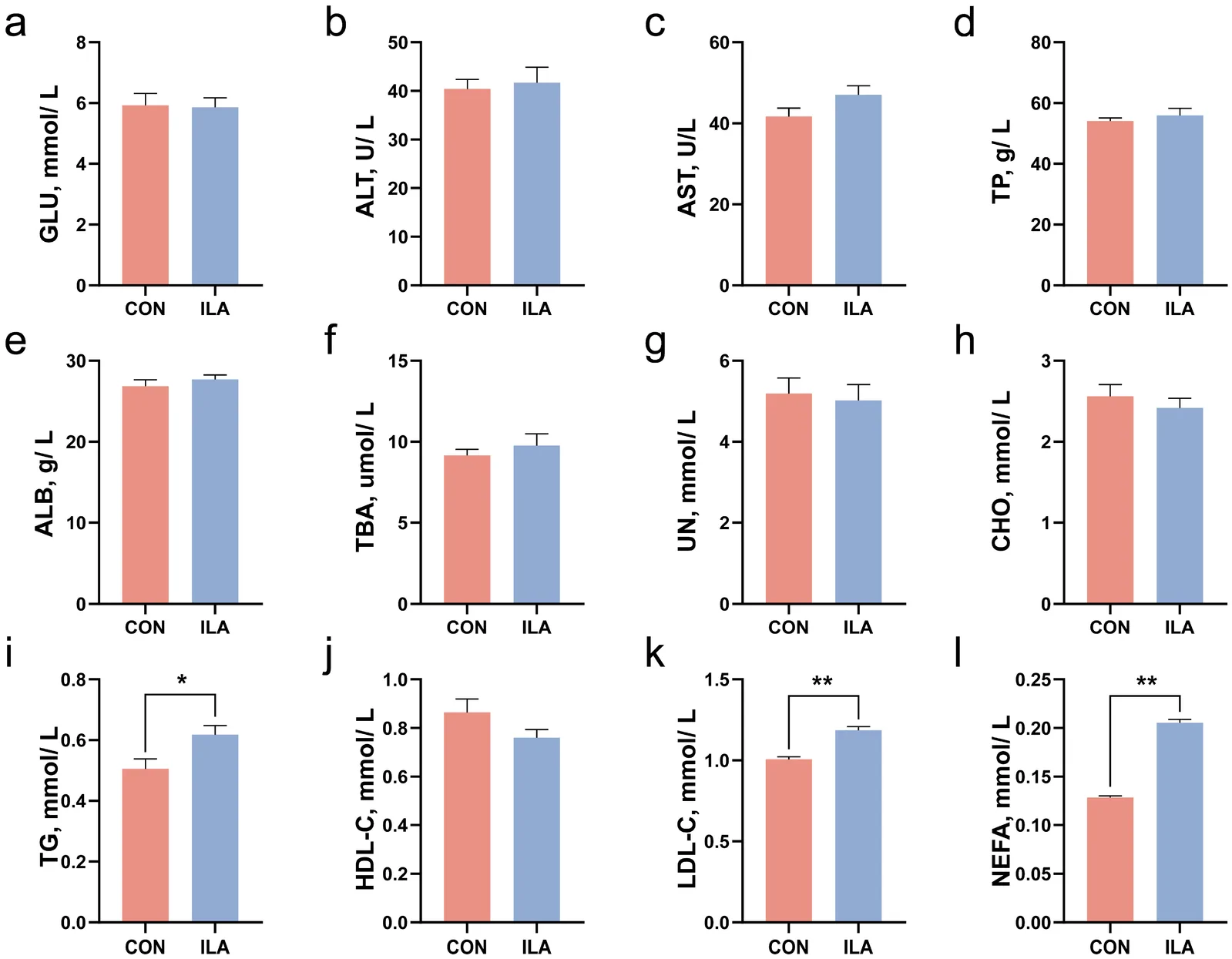
Effects of ILA on serum biochemical parameters (*n* = 6): (**a**) GLU. (**b**) ALT. (**c**) AST. (**d**) TP. (**e**) ALB. (**f**) TBA. (**g**) UN. (**h**) CHO. (**i**) TG. (**j**) HDL-C. (**k**) LDL-C. (**l**) NEFA. Data are presented as the mean ± SEM. * *p* < 0.05, ** *p* < 0.01. Abbreviations: CON, control group; ILA, indole-3-lactic acid; GLU, glucose; ALT, alanine aminotransferase; AST, aspartate aminotransferase; TP, total protein; ALB, albumin; TBA, total bile acids; UN, urea nitrogen; CHO, cholesterol; TG, triglycerides; HDL-C, high-density lipoprotein cholesterol; LDL-C, low-density lipoprotein cholesterol; NEFA, non-esterified fatty acids.

Analysis of serum biochemical profiles revealed that ILA was mainly associated with alterations in lipid metabolism-related parameters. Compared with the control group, serum concentrations of TG, LDL-C, and NEFA were significantly elevated in the ILA group (Figure 2i,k,l). No significant differences were observed in the other measured serum biochemical parameters between the two groups.

### 3.3. ILA Modulated Feeding Behavior

**Figure 3:**
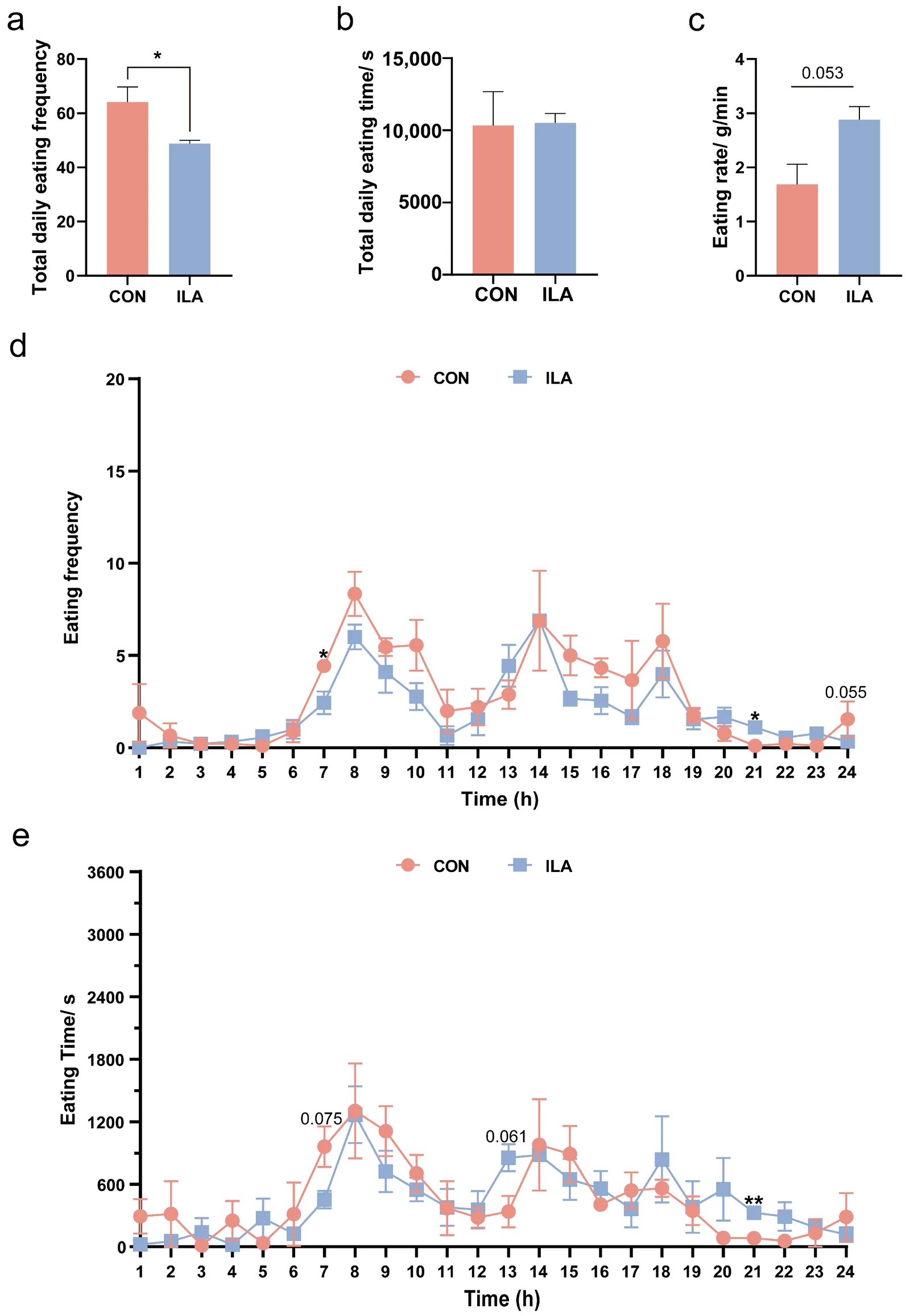
Effects of ILA on eating behavior (*n* = 3): (**a**) Total daily eating frequency. (**b**) Total daily eating time. (**c**) Eating rate. (**d**) The distribution of eating frequency throughout the day. (**e**) The distribution of eating time throughout the day. Data are presented as the mean ± SEM. * *p* < 0.05, ** *p* < 0.01. Abbreviations: CON, control group; ILA, indole-3-lactic acid.

To investigate whether ILA affects feeding behavior, behavioral monitoring was conducted for three consecutive days starting on day 19. Compared with the control group, ILA significantly reduced daily eating frequency (Figure 3a). Further analysis of the 24 h feeding pattern revealed that piglets in the ILA group exhibited a lower eating frequency during the daytime, whereas eating frequency was increased during the early nighttime period compared with the control group (Figure 3d). Although total eating time was not significantly affected by ILA, the temporal distribution of eating time across the 24 h cycle was altered, with increased eating time observed during the early nighttime period in the ILA group (Figure 3b,e). Moreover, ILA tended to increase eating rate (Figure 3c).

### 3.4. ILA Increased Serum Appetite-Related Hormone Levels 

**Figure 4:**
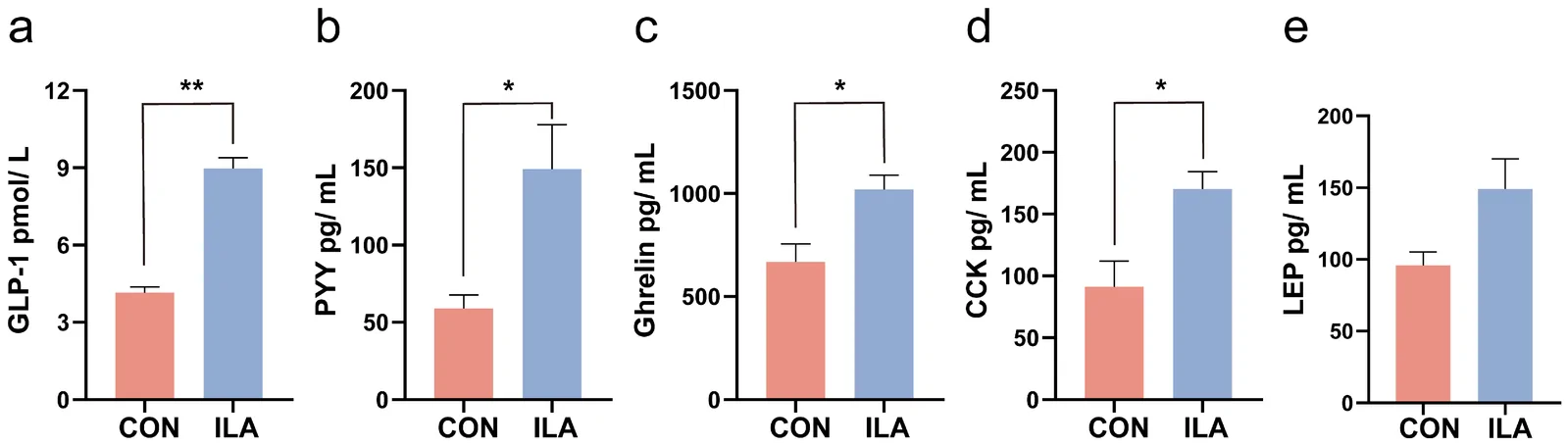
Effects of ILA on serum appetite-related hormone levels (*n* = 6): (**a**) GLP-1. (**b**) PYY. (**c**) Ghrelin. (**d**) CCK. (**e**) LEP. Data are presented as the mean ± SEM. * *p* < 0.05, ** *p* < 0.01. Abbreviations: CON, control group; ILA, indole-3-lactic acid; GLP-1, glucagon-like peptide-1; PYY, peptide YY; CCK, cholecystokinin; LEP, leptin.

Compared with the control group, ILA significantly increased serum levels of GLP-1, PYY, ghrelin, and CCK (Figure 4a–d). In addition, serum levels of LEP tended to increase in the ILA group (Figure 4e).

### 3.5. ILA Altered Hypothalamic Transcription Profile

**Figure 5:**
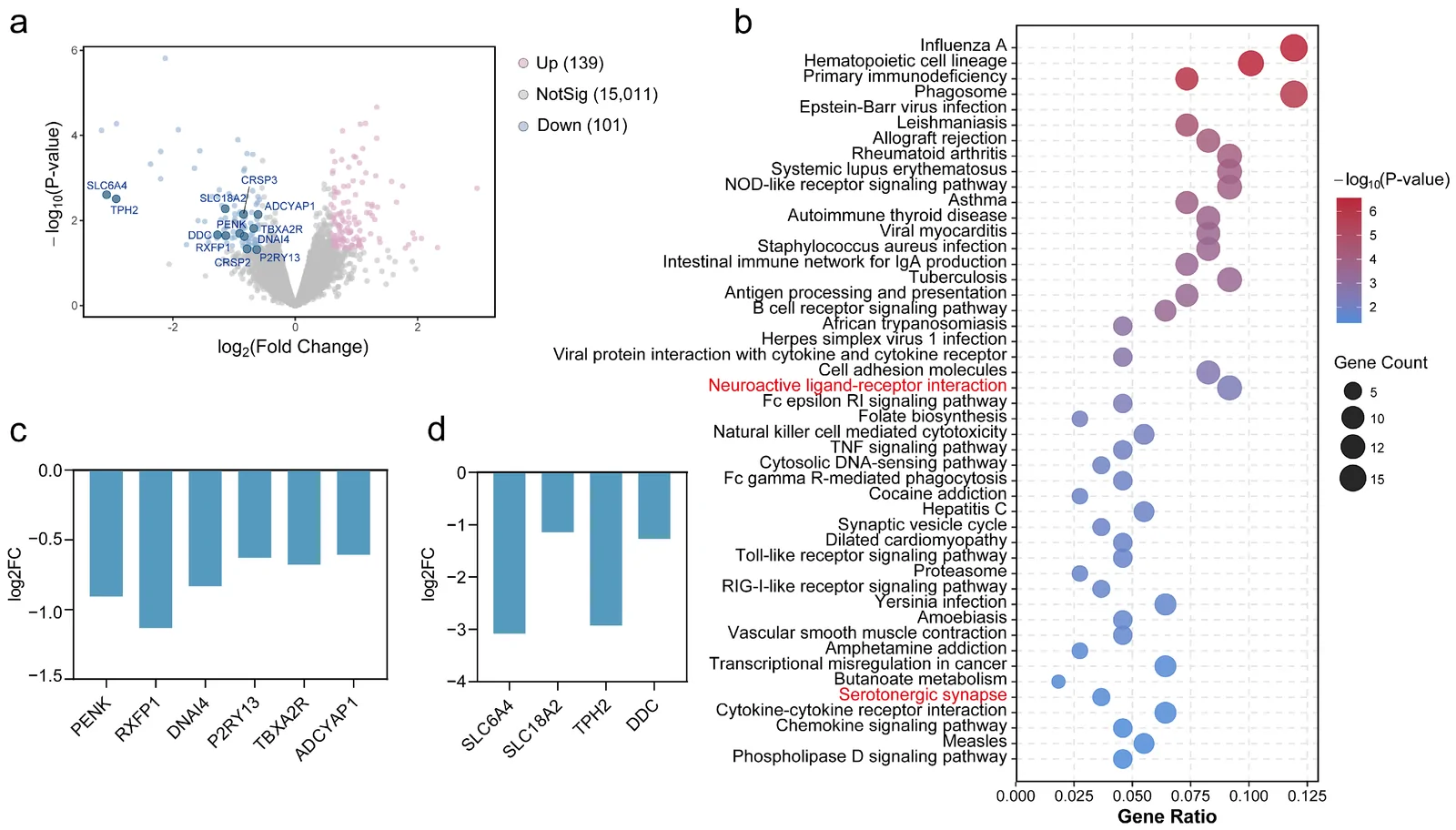
Effects of ILA on hypothalamic transcription profile (*n* = 4): (**a**) Volcano plot showing DEGs between the ILA and control groups. (**b**) KEGG pathway enrichment analysis of DEGs. (**c**) Expression of DEGs associated with the neuroactive ligand–receptor interaction pathway. (**d**) Expression of DEGs associated with the serotonergic synapse pathway.

To further investigate hypothalamic transcriptional responses associated with ILA treatment in feeding regulation, a hypothalamic transcription profile was performed. It turned out that ILA upregulated 139 genes and downregulated 101 genes compared to the control group (Figure 5a). KEGG pathway enrichment analysis showed that the DEGs were enriched in several pathways. Among these pathways, the serotonergic synapse and neuroactive ligand–receptor interaction pathways were particularly noteworthy due to their potential roles in neural signaling and feeding regulation (Figure 5b). Further analysis showed that all pathway-associated DEGs identified in the neuroactive ligand–receptor interaction and serotonergic synapse pathways were downregulated in the ILA group (Figure 5c,d). These included genes associated with serotonergic neurotransmission, such as SLC6A4, SLC18A2, TPH2, and DDC, as well as genes potentially involved in appetite regulation, including PENK, ADCYAP1, and RXFP1.

### 3.6. Correlation Analysis of Hypothalamic Appetite-Related Genes with Feeding-Related Traits

**Figure 6:**
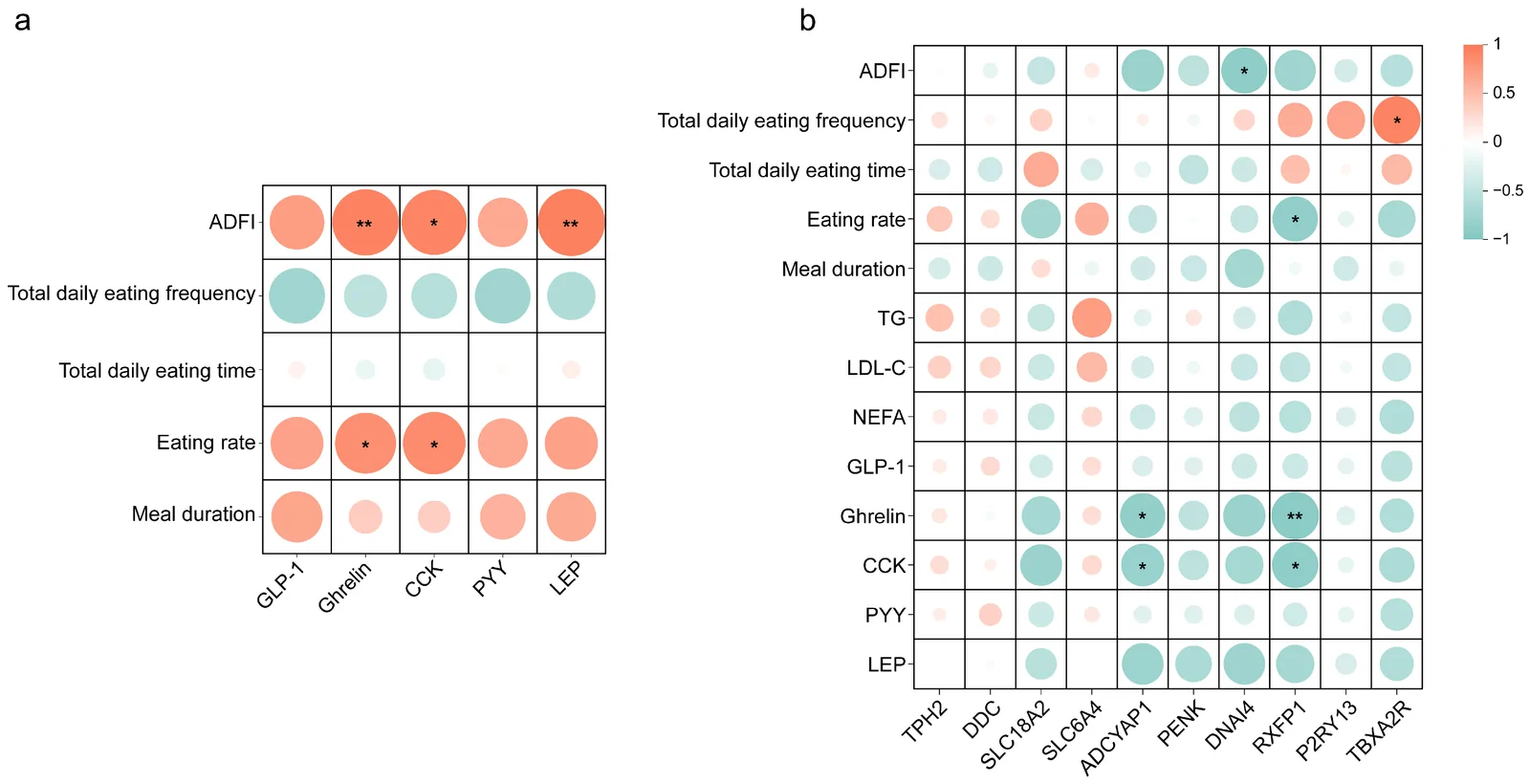
Correlation analyses of feeding behavior, serum parameters, and hypothalamic appetite-related gene expression in weaned piglets (*n* = 3 per group): (**a**) Correlation analysis between feeding behavior parameters and serum appetite-related hormone levels. (**b**) Correlation analysis between hypothalamic appetite-related genes and growth performance, eating behavior, serum biochemical profiles, and appetite-related hormones. * *p* < 0.05; ** *p* < 0.01.

Spearman correlation analysis was performed to evaluate the associations between feeding behavior parameters and serum appetite-related hormone levels, as well as between hypothalamic appetite-related gene expression and feeding-related traits. As shown in Figure 6a, ADFI was positively correlated with serum ghrelin, CCK, and leptin levels (*p* < 0.05). Eating rate was also positively correlated with serum ghrelin and CCK levels (*p* < 0.05). No significant correlations were observed between the other examined feeding behavior parameters and serum appetite-related hormone levels. As shown in Figure 6b, the expression of several hypothalamic appetite-related genes was significantly correlated with feeding-related traits and serum hormone levels. Specifically, ADCYAP1 expression was negatively correlated with serum ghrelin and CCK levels (*p* < 0.05). RXFP1 expression was negatively correlated with eating rate, serum ghrelin, and CCK levels (*p* < 0.05). In addition, DNAI4 expression was negatively correlated with average daily feed intake (ADFI), whereas TBXA2R expression was positively correlated with total daily eating frequency (*p* < 0.05).

## 4. Discussion

Gut microbiota-derived metabolites have recently been recognized as important signaling molecules linking the gut microbiota to host physiological functions and have been implicated in the regulation of host metabolism and behavior [12]. In our previous study, short-term supplementation with ILA resulted in a numerical increase in average daily feed intake and tended to increase circulating GLP-1 levels in piglets. Therefore, the present study investigated the effects of long-term dietary ILA supplementation on growth performance, feeding behavior, and appetite-related signaling in weaned piglets. Our results showed that although ILA did not significantly alter growth performance, average daily feed intake was numerically higher in the ILA group, feeding behavior patterns were modified, and serum lipid metabolism-related parameters and multiple appetite-related hormones were altered. Furthermore, ILA altered the expression of hypothalamic genes potentially involved in appetite regulation.

Feeding behavior during the early post-weaning period affects not only nutrient intake and growth performance but is also closely associated with the development of intestinal function, immune homeostasis, and metabolic adaptation [26,27,28]. Feeding behavior is a complex physiological process that extends beyond total feed intake and includes multiple dimensions, such as eating frequency, eating time, eating rate, and diurnal feeding patterns. These behavioral parameters collectively reflect the integration of energy requirements, nutrient availability, and satiety-related signals. In the present study, long-term ILA supplementation did not significantly affect growth performance in weaned piglets, although BW, ADG, and ADFI were numerically higher in the ILA group than in the control group. Previous studies have shown that different tryptophan metabolites can influence growth performance in weaned piglets. For example, dietary supplementation with 5-hydroxytryptophan increased ADG and improved feed efficiency, whereas dietary supplementation with indole-3-propionic acid increased ADG during certain periods [29,30]. Although ILA supplementation did not significantly improve growth performance in the present study, the numerical increases in BW, ADG, and ADFI were consistent in direction with findings from studies of other tryptophan metabolites. In the present study, ILA significantly reduced daily eating frequency in weaned piglets, whereas total eating time was not affected and eating rate tended to increase. These findings suggest that ILA may alter feeding patterns by reducing feeding frequency without significantly affecting total feeding time. Pigs exhibit considerable flexibility in feeding behavior and can adjust eating frequency, eating time, and eating rate in response to changes in nutritional conditions or feeding strategies, thereby helping to enhance nutrient intake and meet the requirements for maintenance and growth [31,32,33]. Feeding behavior in pigs exhibits distinct diurnal patterns and is jointly regulated by environmental factors, nutritional status, and metabolic signals [34,35,36]. In this study, ILA altered the distribution of feeding behavior over the 24 h period, with lower eating frequency during the daytime and higher eating frequency and eating duration during the early nighttime period. Similarly, previous studies showed that dietary tryptophan supplementation reduced morning feeding duration in piglets without affecting overall feed intake [37]. These findings suggest that ILA may influence the temporal organization of feeding behavior rather than simply altering total feed consumption. Furthermore, serum biochemical analysis showed that ILA was associated with changes in lipid metabolism-related parameters. Compared with the control group, serum TG, LDL-C, and NEFA levels were significantly increased in the ILA group. NEFA is an important indicator of lipid mobilization, and its elevation generally reflects increased fatty acid release from adipose tissue to provide substrates for energy metabolism. The higher TG and LDL-C levels also suggest that ILA may affect lipid metabolism. Taken together, these findings suggest that changes in feeding behavior following ILA supplementation may be accompanied by changes in lipid metabolism. However, because growth performance did not differ significantly between the groups, the relationship between these behavioral and metabolic changes and the maintenance of growth performance remains unclear.

From an animal production perspective, the present findings provide preliminary evidence that ILA may serve as a nutritional modulator of feeding behavior in weaned piglets. Feeding behavior is an important consideration in pig production, particularly around weaning, when feed intake and adaptation to solid diets are closely related to piglet performance and welfare [38]. Previous research has shown that feeding regimens can alter eating frequency and eating duration, with corresponding changes in growth performance, although feed efficiency may not be affected [39]. In the present study, ILA supplementation reduced daily eating frequency without significantly affecting total daily eating time, average daily feed intake, or growth performance. These findings suggest that ILA may influence feeding patterns without necessarily changing overall feed consumption under the conditions investigated. However, whether such changes confer practical benefits, such as improved feed efficiency, growth performance, or post-weaning adaptation, remains unclear and requires further investigation under commercial production conditions.

Gastrointestinal hormones serve as important signaling molecules linking intestinal nutrient sensing with central appetite regulation and play a critical role in maintaining feeding behavior homeostasis [40]. To further investigate the potential peripheral responses to ILA supplementation, the levels of major appetite-related gastrointestinal hormones in serum were determined. The results showed that ILA significantly increased serum levels of GLP-1, CCK, PYY, and ghrelin. Notably, ghrelin is generally elevated in response to increased energy demands, whereas satiety-related hormones, including GLP-1, CCK, and PYY, typically remain at relatively low levels. However, in the present study, ILA simultaneously enhanced both hunger-related and satiety-related signals, suggesting that ILA may influence feeding behavior through the combined actions of multiple gastrointestinal hormones rather than through a single regulatory pathway [41]. The hormonal changes may have opposing effects on feeding, which could help explain why total feed intake was not significantly altered despite the marked changes in gastrointestinal hormone levels. Ghrelin primarily contributes to the initiation of feeding motivation during energy deficiency, whereas GLP-1, CCK, and PYY convey postprandial nutrient status signals and participate in satiety regulation. Therefore, the concurrent increase in ghrelin and satiety-related hormones may reflect a more complex alteration in peripheral appetite-related signaling rather than a unidirectional change in appetite. This overall change in the gastrointestinal hormone profile may be associated with the altered feeding pattern observed in the ILA group, suggesting that ILA may influence feeding behavior through the coordinated regulation of multiple appetite-related hormonal signals rather than by exerting a simple stimulatory or inhibitory effect on feed intake.

Gastrointestinal hormones are mainly secreted by enteroendocrine cells (EECs), and the formation and maintenance of EECs depend on continuous renewal and differentiation of intestinal epithelial stem cells [42,43]. Previous studies have demonstrated that the microbiota-derived tryptophan metabolite ILA can regulate intestinal epithelial homeostasis and promote intestinal stem cell proliferation and differentiation [44,45]. In addition to these effects, microbial tryptophan metabolism has been implicated in the maintenance of intestinal barrier integrity and mucosal immune homeostasis. Disruptions in these processes may contribute to increased intestinal permeability and inflammatory responses, highlighting the broader relevance of tryptophan metabolism to gut health and host physiology [15,16]. In addition, our previous study showed that ILA promotes the differentiation of porcine intestinal stem cells toward the EEC lineage and enhances the expression of the L-cell marker gene Gcg and GLP-1 secretion [24]. These findings provide a possible basis for the alterations in gastrointestinal hormones observed in the present study. However, intestinal endocrine function was not directly examined in the present study, and the relationship between changes in intestinal endocrine function and the altered feeding phenotype remains unclear. Collectively, these findings suggest that changes in appetite-related gastrointestinal hormone levels may be associated with the altered feeding behavior following ILA supplementation. However, the underlying physiological relationships require further investigation.

In the correlation analysis, serum ghrelin and CCK levels were positively associated with both ADFI and eating rate. These associations suggest that ghrelin and CCK may be relevant to individual variation in feeding-related traits in the present study. Previous research in grow-finish pigs reported associations between circulating acyl-ghrelin concentrations and feeding behavior, including daily meal frequency and meal duration [46]. This provides support for a potential relationship between ghrelin signaling and feeding patterns in pigs. However, CCK is generally recognized as a satiety-related hormone that contributes to meal termination [47]. Therefore, the positive correlations of CCK with ADFI and eating rate in the present study should not be interpreted as evidence that CCK directly stimulates feed intake. Rather, these associations may reflect the complexity of peripheral hormonal responses accompanying feeding behavior. Given the limited sample size, these correlations should be interpreted cautiously and do not establish a causal relationship between hormonal changes and feeding behavior.

Peripheral nutrient signals are integrated by the central nervous system to regulate feeding behavior. The hypothalamus, as a key brain region involved in the regulation of energy homeostasis and feeding behavior, integrates metabolic signals from the periphery and participates in feeding regulation through modulation of neurotransmitters, neuropeptides, and related signaling pathways. Therefore, we performed hypothalamic transcriptomic analysis to investigate transcriptional changes associated with ILA supplementation. The present study showed that ILA significantly downregulated the expression of multiple genes associated with serotonergic signaling, including TPH2, DDC, SLC6A4, and SLC18A2, which are collectively involved in serotonin synthesis, transport, and reuptake [48,49,50,51]. The hypothalamic serotonergic system contributes to satiety signaling and feeding regulation, in part through its effects on POMC and AgRP/NPY neuronal activity [52]. Therefore, the reduced expression of these serotonin-related genes may be associated with changes in hypothalamic satiety signaling. In addition, ILA downregulated the expression of several neuropeptide- and neuroendocrine-related genes, including ADCYAP1 and PENK. ADCYAP1 encodes pituitary adenylate cyclase-activating polypeptide (PACAP), which is involved in hypothalamic regulation of energy homeostasis and feeding behavior [53,54]. In the present study, ADCYAP1 expression was negatively correlated with serum ghrelin and CCK levels, suggesting a possible link between PACAP-related neural signaling and peripheral gastrointestinal hormone levels. Previous studies have shown that exogenous ghrelin can influence PACAP-related signaling, further supporting a potential interaction between peripheral energy status signals and PACAP-mediated neuroendocrine pathways [55]. Furthermore, PENK, a component of the endogenous opioid system, is widely expressed in multiple regions of the central nervous system and is involved in feeding motivation, food reward, and emotion-related behaviors. Unlike homeostatic feeding regulation, which primarily reflects physiological energy requirements, the opioid system is more closely involved in food palatability and reward processing [56]. Previous studies have shown that PENK deficiency reduces the intake of highly palatable foods, such as high-sugar and high-fat diets, indicating that PENK signaling contributes to the regulation of food reward value and feeding drive [57]. In the present study, the downregulation of PENK expression suggests that ILA may reduce reward-related feeding motivation. Since the reward system enhances responses to food-related stimuli and promotes additional food intake beyond energy requirements, reduced PENK signaling may weaken the influence of food-related cues on feeding initiation, thereby reducing frequent feeding events driven by food reward. Combined with the increased levels of satiety-related gastrointestinal hormones, including GLP-1, CCK, and PYY, ILA may simultaneously enhance postprandial satiety feedback. Therefore, changes in PENK expression and gastrointestinal hormone levels may be associated with the reduction in eating frequency in ILA-treated piglets. However, further studies are needed to determine whether these changes directly contribute to the observed behavioral effects.

Several limitations should be acknowledged in the present study. First, the relatively low initial body weight of the piglets may limit the generalizability of our findings to pigs with different initial body weights or at other developmental stages. Body weight and developmental stage may influence feeding behavior, metabolic status, and circulating appetite-related hormone levels, and thus should be considered when interpreting the present results. Further studies involving piglets with different initial body weights and at different developmental stages are needed to determine whether these factors modify the effects of ILA. Second, gastrointestinal hormone levels were measured at a single time point, limiting our ability to characterize the changes in ghrelin, GLP-1, CCK, and PYY across the fasting–feeding cycle and to determine their temporal relationship with ILA-induced changes in feeding behavior. Third, although hypothalamic transcriptomic analysis identified changes in expression of genes and in signaling pathways associated with feeding regulation, the functional significance of these changes and their direct role in the feeding of ILA remain unclear. Finally, our previous findings showed that ILA can influence intestinal enteroendocrine cell-related processes; however, whether these effects are associated with alterations in gastrointestinal hormone signaling in the present model remains unclear. Further studies are therefore needed to determine whether intestinal endocrine responses contribute to the effects of ILA on feeding behavior.

## 5. Conclusions

This study investigated the effects of ILA on growth performance, feeding behavior, and appetite-related signals in weaned piglets. ILA did not significantly affect growth performance, although numerical increases in final body weight, average daily gain, and average daily feed intake were observed. ILA altered feeding patterns, characterized by a reduced daily feeding frequency and an increased feeding rate, without significantly changing total feeding time. ILA also altered appetite-related gastrointestinal hormones, including GLP-1, ghrelin, CCK, and PYY. Hypothalamic transcriptomic analysis further revealed that ILA altered genes and signaling pathways associated with feeding regulation, particularly neurotransmitter-related pathways. In summary, ILA modulated feeding behavior and was accompanied by changes in peripheral appetite-related signals and hypothalamic transcriptional responses in weaned piglets, suggesting a potential role of ILA in feeding regulation.

## Figures and Tables

**Table 1:** Composition and nutrient level of the basal diet (as-fed basis).

Ingredient	Percentage (%)	Calculated Nutritional Compositions (%)	
Corn	60.65	Digestible energy (MJ/kg)	14.2
Soybean meal	15.00	Crude protein (%)	17.8
Extruded soybean	8.00	Lysine (%)	1.30
Imported fish meal	2.00	Methionine + cystine (%)	0.70
Wheat bran	5.00	Threonine (%)	0.75
Soybean protein concentrate	2.00	Tryptophan (%)	0.23
Low-protein whey powder	2.50		
Sucrose	2.00		
Soybean oil	0.60		
L-Lysine HCl (98.0%)	0.45		
DL-Methionine (99%)	0.10		
L-Threonine (98.5%)	0.05		
L-Tryptophan (98%)	0.02		
L-Valine (98%)	0.08		
stone powder	0.20		
Dicalcium phosphate	0.15		
Zinc oxide (80%)	0.20		
Choline chloride (50%)	0.20		
Sodium chloride	0.30		
Piglet premix	0.50		
Total	100.00		

## Data Availability

The data presented in this study are available in the article. Further inquiries can be directed to the corresponding author.
